# High Figure of Merit Optical Buffering in Coupled-Slot Slab Photonic Crystal Waveguide with Ionic Liquid

**DOI:** 10.3390/nano10091742

**Published:** 2020-09-03

**Authors:** Israa Abood, Sayed Elshahat, Zhengbiao Ouyang

**Affiliations:** 1 College of Physics and Optoelectronic Engineering, Shenzhen University, Shenzhen 518060, China; i.abood@szu.edu.cn; 2 THz Technical Research Center of Shenzhen University, Shenzhen. Key Laboratory of Micro-nano Photonic Information Technology, Key Laboratory of Optoelectronics Devices and Systems of Ministry of Education and Guangdong Province, Shenzhen University, Shenzhen 518060, China; selshahat@szu.edu.cn; 3 Institute of Microscale Optoelectronics, Shenzhen University, Shenzhen 518060, China; 4 Physics Department, Faculty of Science, Assiut University, Assiut 71516, Egypt

**Keywords:** slow light, buffering capability, photonic crystal waveguide, ionic liquids

## Abstract

Slow light with adequate low group velocity and wide bandwidth with a flat band of the zero-dispersion area were investigated. High buffering capabilities were obtained in a silicon-polymer coupled-slot slab photonic crystal waveguide (SP-CS-SPCW) with infiltrating slots by ionic liquid. A figure of merit (FoM) around 0.663 with the lowest physical bit length $L_{bit}$ of 4.6748 µm for each stored bit in the optical communication waveband was gained by appropriately modifying the square air slot length. Posteriorly, by filling the slots with ionic liquid, the $L_{bit}$ was enhanced to be 4.2817 μm with the highest FoM of 0.72402 in wider transmission bandwidth and ultra-high bit rate in terabit range, which may become useful for the future 6G mobile communication network. Ionic liquids have had a noticeable effect in altering the optical properties of photonic crystals. A polymer was used for the future incorporation of an electro-optic effect in buffers to realize the dynamic controlling of optical properties. Ionic liquids enhanced the transmission rate through optical materials. Additionally, the delay time in the ns-range was achieved, providing longer delay and ultra-low group velocity, which is important for light-matter interaction in light amplifiers and nonlinear devices.

## 1. Introduction

Incoming polymer technology has provided attractive candidates for photonic materials due to their low-cost production and wide bandwidth [1,2,3]. Wide bandwidth, dispersion-free propagation in a photonic crystal waveguide (PCW) is a worthy nominee for slow-light implementation, which reinforces the trapping and guiding of optical pulses. Slow light encourages stronger light-matter interaction while providing additional control over the spectral bandwidth for this interaction and allowing delaying and temporarily storing of light [4]. Room temperature ionic liquids (RTILs) are carbon-based salts that are in a fluid state at room temperature. As a new polar solvent, ionic liquid has almost no vapor pressure, is nonvolatile and noninflammable, and has a low melting point below 100 °C [5]. RTILs possess strong electrostatic field, good electrical and thermal conductivity, good light transmittance and high refractive index [6,7], good chemical stability and thermal stability, and are recyclable and environmentally friendly. So, it is called “green” chemical solvent to replace the traditional volatile toxic solvent [8,9]. Therefore, ionic liquids are considered as one of the most promising green solvents and catalysts in the 21st century and have been applied in many fields such as biocatalysis, separation technology, and electrochemistry [10]. Furthermore, some polymerized RTILs can work as the final material for passive optical components. Such material can combine the benefits of polymers with that of dyes and nanoparticles. Polymerized RTILs are incompletely transparent in the visible waveband and completely transparent in the near-IR. Several RTILs with relatively low indices $\left(n_{f}<1.50\right)$ and also high-index RTILs ($\left(n_{f}>1.70\right)$ are commercially available [5]. These ILs are used as immersion liquids to tune the optical properties of photonic crystals (PhCs) or to match the refractive indices of the constituting materials of these photonic crystals. Moreover, the refractive indices of polymerized RTILs have been determined because they are important for producing microstructures in photonics. Their refractive index is about 1.6–1.7 in the wavelength range of 600–2000 nm for both polymerized RTILs, which include optical communication wavelength 1550 nm evaluated based on ellipsometry measurements [11]. From the optical properties of polymerized RTILs, they seem to be promising materials for the fabrication of infrared photonic elements. The RTIL-based planar optical components can be realized on glass substrates because of the relatively high refractive indices of RTIL-derived polymers (about 1.65) [5]. The potential applications for micro-optical components include waveguides, micro-resonators, and photonic crystals, which are essential components in photonic integrated circuits (PICs) [12]. Different microfabrication techniques have been applied in the technology of polymer-based micro-optical devices, including electron beam lithography (EBL) and soft lithography [13] or electron beam etching [14]. Lately, RTIL is used instead of polymers as an electron beam (EB)-sensitive material [12,15]. The conditions of the fabrication would be chosen carefully to minimize the fabrication errors of the designed parameters. Therefore, it is necessary to use some processes with high precision, such as high-resolution lithography and high-quality pattern. Thus, the direct writing method by electrons is attractive, particularly in terms of obtaining high resolution without using additional etching steps or photolithography masks [12]. Consequently, we considered polymerized RTILs in our calculations based on silicon-polymer, coupled-slot, slab photonic crystal waveguide (SP-CS-SPCW), which exploits the benefits of both coupled-slot cavity and RTILs infiltrated.

## 2. Theoretical Characteristics

The performance and properties of slow light are often specified with more significant parameters. The first is the group velocity of guided mode, which is obtained by the well-known dispersion relationship:

(1) $$v_{g}=\;\frac{d\omega }{dk}=\;\frac{c}{n_{g}},$$

where ω and k are the angle frequency and wavevector of the incident light, c is the speed of light in free space, and $n_{g}$ is the group index inside the PhC medium. The second is the group-velocity dispersion (GVD, or $\beta_{2}$):

(2) $$GVD=\beta_{2}=\;\frac{d^{2}k}{d\omega^{2}}=\frac{a}{2\pi c^{2}}\frac{dn_{g}}{dU}.$$

where U=ωa/2πc is the normalized frequency and *a* is the lattice constant. The third is the normalized delay bandwidth product (NDBP), which is the most important parameter for slow-light performance. It can be calculated as the multiplication of the average group index ${\bar{n}}_{g}$ per frequency range, where ${\bar{n}}_{g}$ is restricted by over ±10% variation of group index [16,17] and the bandwidth ratio $\left(∆U/U_{0}\right)\;$ [18]:

(3) $$NDBP=\;{\bar{n}}_{g}\;\times \;\frac{∆\omega }{\omega_{0}}=\;{\bar{n}}_{g}\;\times \;\frac{∆U}{U_{0}}.$$

where ∆ω and $\omega_{0}$ are the bandwidth and central frequency, respectively; ∆U=∆ωa/2πc and $U_{0}=\omega_{0}a/2\pi c$ are the normalized bandwidth and normalized central frequency, respectively. NDBP is an indispensable quantity in representing the slow-light performance and more often used when the devices have different lengths and/or operating frequencies [19,20,21,22]. 

Buffering performance includes several parameters related to dispersion curves: Storage time $T_{s}$ in the waveguide, physical bit length $L_{bit}$, buffer capacity C, and bit rate $R_{b}$. The optical buffer is used to store data and adjust the timing of optical packets. For the PhC slab waveguide length of L, the storage time can be obtained by $T_{s}=\;L/v_{g}$. The bit rate can be calculated by [23]

(4) $$R_{b}=B_{packet}/2=\;\frac{c\;\times \;∆U}{4a},$$

where $B_{packet}$ is the baseband bandwidth of the input data. It is important to find the balance between device bit length and bit rate. The average buffer capacity (C) can be calculated by

(5) $$C=\;T_{s}\;B_{packet}=\;\frac{L\times {\bar{n}}_{g}}{c}\;\times \frac{c\;\times \;∆U}{2a}=\frac{LU_{0}}{2a}\;\times NDBP.$$

For all delay-line buffers, the minimum physical size of the buffer for a given number of buffered data bits is ultimately limited by the physical size of each stored bit. All slow-light optical buffers require a lower value of the stored bit length. The materialistic size of a bit stored, the bit length, can be calculated as [24]:

(6) $$L_{bit\;}=\frac{L}{C}=\;\frac{2a}{U_{0}\times NDBP}.$$

From Equations (5) and (6), the remarkable figure of merit (FoM) for all slow-light devices and an optical buffer is *NDBP*, which requires a lower value of the stored bit length. 

## 3. Structure Geometry and Simulation Results 

**Figure 1:**
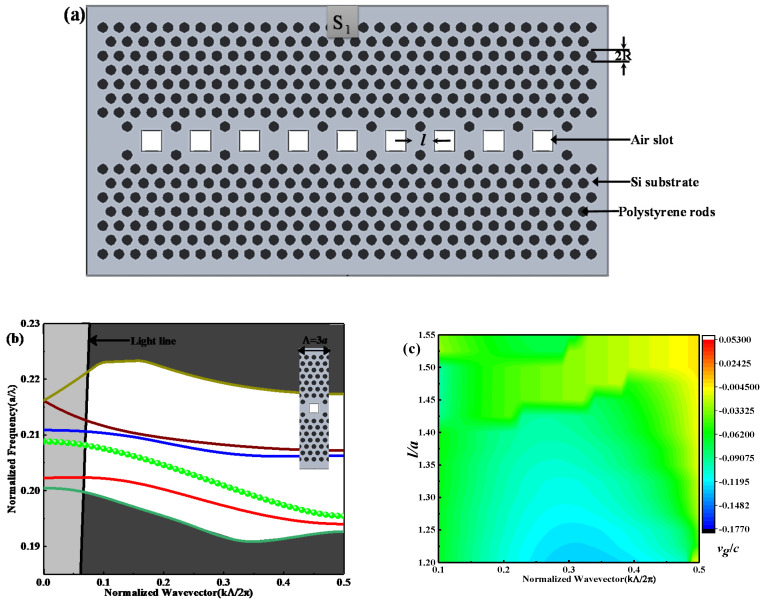
(**a**) Schematic structure of the proposed S1 SP−CS−SPCW geometry based on triangular-lattice photonic crystal made up of holes filled with electro-optic polystyrene (refractive index $n_{poly}=1.59$) with basic hole radius R=0.30a in a silicon substrate of $n_{si}=3.48$; (**b**) the dispersion curve for TM with l=1.30a, where the inset shows the supercell used for simulation; (**c**) the normalized-group velocity ($v_{g}/c$) contour map with l varying from 1.20a to 1.55a via normalized wavevector K.

The proposed SP-CS-SPCW structure is based on a triangular lattice photonic crystal made from a silicon substrate with refractive index $n_{si}=3.48$ and basic holes filled with highly nonlinear poled electro-optic polystyrene of $n_{poly}=1.59$, with elementary radius R=0.30a. The coupled-slot cavity waveguide S1 is first constituted by inserting square air slots of length l into the center of the waveguide, as shown in Figure 1a. Afterward, the square air slots will be filled by the ionic liquid. The spacing between each two neighboring square slots is 3a. Suppose, for example, that the dielectric constant is independent on the Y-axis. At that point, the solutions of all take the form of either transverse magnetic (TM) modes with nonzero $\left(H_{y},\;E_{x},\;E_{z}\right)$ (H field refer to Magnetic component and E field refer to Electric component.) or transverse electric (TE) modes with nonzero $\left(H_{x},\;H_{z},\;E_{y}\right)$. Our proposed structures support TM only. Figure 1b displays the dispersion curve for TM mode of the square slot defect at l=1.30a of S1, i.e., the first derivative between the normalized frequency U=ωa/2πc=a/λ and the normalized wavevector K=ka/2π for the dispersion relation of the guided mode. The slope of the dispersion curve gives $n_{g}$ that is acquired from Equation (1) by the first derivative of the dispersion curve. Accordingly, from $n_{g}$ Equation and dispersion curve, as shown in Figure 1b, when *U* is almost unchanged it refers to a flat area of guided mode with changing K, $n_{g}$ going to ultra-high value or infinity, then going to slow-light flat band with a constant slope, which refers to constant $n_{g}$ during changing *U* with *K*. S_1_ SP-CS-SPCW maintained multi-modes inside the Photonic Band Gap (PBG). In this study, we were concerned with the green-guided mode, due to its wide transmission bandwidth. The dispersion curves in Figure 1b were obtained by the plane-wave expansion method (PWE) with the BandSOLVE module of software Rsoft and the inset of Figure 1b is the supercell selected for eigenmode calculations. The solid black line indicates the light line. Figure 1c shows the group velocity $v_{g}/c$ contour map with l varying from 1.20a to 1.55a via normalized wavevector K=kΛ/2π. We can see that the linearity at the long *K* range decreases with increasing l until l=1.40a but with high group velocity value, namely low group index. 

**Figure 2:**
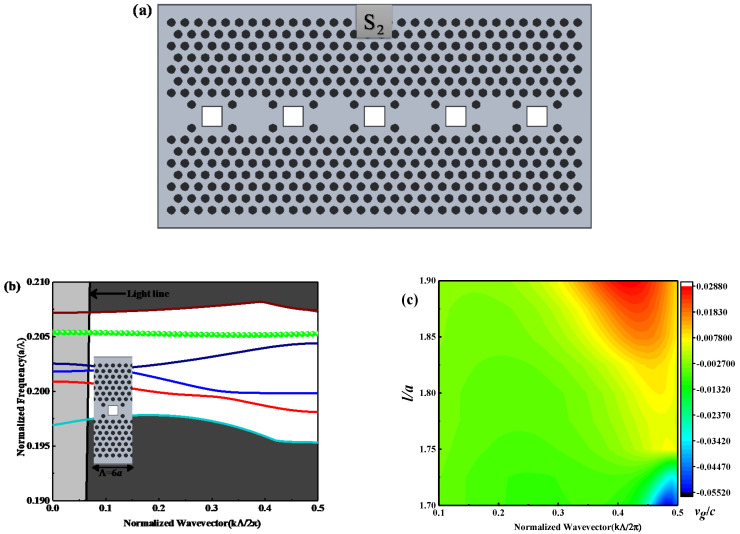
(**a**) Schematic structure of the proposed S2 SP-CS-SPCW geometry; (**b**) the dispersion curve for TM with l=1.80a, where the inset shows the supercell used for simulation; (**c**) the normalized group velocity $\left(v_{g}/c\right)$ contour map with l varying from 1.70a to 1.90a via normalized wavevector K.

**Figure 3:**
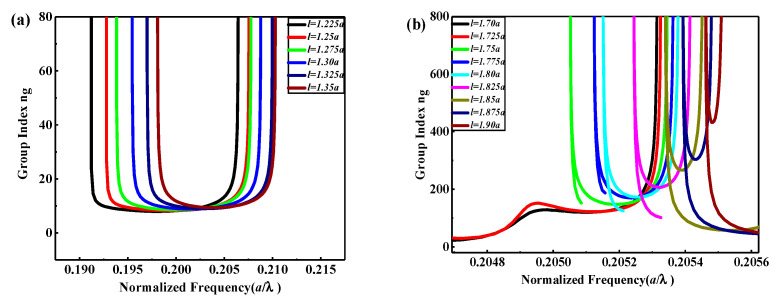
The group index ($n_{g})$ versus normalized frequency a/λ with changing defect length l for (**a**) S_1_ and (**b**) S_2_.

For the comprehension of slow-light devices, the dispersion curves of guided modes should be below the light line, as the guided modes above the light line are lossy and those under the light line are intrinsically lossless [25]. For near-zero group velocity, a high group index S_2_ SP-CS-SPCW is presented by removing one square slot and remains the other, to form the coupling between slab and slot for increasing the quality factor of each slot cavity and, thus, the filed localization inside the cavity, as shown in Figure 2a. Hence, the spacing between each two neighboring square slot cavities is 6a. Accordingly, the width of the supercell, as shown by the inset of Figure 2b, becomes 6a. Figure 2b displays the dispersion curve for TM mode. Figure 2c shows the square slot defect of l=1.80a with a flat band for the normalized group velocity $\left(v_{g}/c\right)$ with l varying from 1.70a to 1.90a. Besides, for additionally indistinct observation for the transmission bandwidth and group index variations, Figure 3a,b shows the variation of $n_{g}$ with normalized frequency for TM modes as l changing from 1.225a to 1.35a and 1.70a to 1.90a, respectively, with an increment of ∆l=0.025a. It is obvious from Figure 3 that the performance of the guided modes of slow light is significantly affected by the geometrical length of the square slot. The guided modes of the slow light shift in the two structures, S_1_ and S_2,_ to the higher frequency area as the slot length increases. This is physically understandable. As the length increases, the effective refractive index of the structure decreases, so that the optical size of the structure decreases and, thus, the frequency response scales to the high-frequency area. 

From Figure 3a, we can obtain a wider bandwidth with a closely constant dispersion curve slope, i.e., flat band of zero-dispersion area for large NDBP and high-capability optical buffering but with low $n_{g}$. On the other hand, higher $n_{g}$ can be obtained from Figure 3a with narrower transmission bandwidth. To analyze the data results theoretically, we transferred the results in Figure 3 into Table 1. Based on the PWE method, which deals with infinite structure area, a longer delay-line length *L* is taken as 1 mm by cascading the unit cell structure shown in Figure 1b and Figure 2b, which is much longer than the wavelength. So, our calculations are applicable [17]. The operating bandwidth was calculated for within ±10% variation of $n_{g}$ as the operating wavelength changes. The lattice constant *a* is changed to acquire a center wavelength of 1550 nm. It should be noted that, with the scaling characteristic of PhCs, the relationship between the operational wavelength λ and the lattice constant a can be expressed as [26] $a=U_{0}\lambda_{0}$, where $U_{0}$ is the central normalized measured frequency for each given $n_{g}$. Therefore, the properties of PhC can remain unchanged, provided one expands or decreases the parameters of the PCW structure proportionally. One can select the value of the optimum operating wavelength of the structure by only setting the value of a without repeating the simulation [25]. In practical application, the PhC device can be designed according to the demand of the delay time and storage capacity. For better showing the improvement of slow-light property, the corresponding buffering parameters are also shown in Table 1. It can be seen from Figure 3 and Table 1 that, for S_1_ at l = 1.225 *a*, the highest bandwidth with a zero-dispersion flat band was obtained at about ∆λ = 104.81 nm, however, with the lowest average group index of ${\bar{n}}_{g}\;$= 9.5, and the storage time was the lowest at about $T_{s}$= 31.667ps. According to Equation (4), it corresponds to a high bit rate in the range of terabit about $R_{b}=3.2718\;\mathrm{Tb}/s$, which is suitable for signal transmission application, whereas it is preferable for signal buffering at l=1.25a, which corresponds to the highest FoM, i.e., *NDBP* = 0.66313, which is the highest compatible value between ${\bar{n}}_{g}$ and ∆U. A high storage capacity *C* of 213.912 bit along 1-mm delay-line length is obtained with the delay time in picosecond range. This case maybe become useful for the future 6G mobile communication network due to its highest $R_{b}$ with the smallest $L_{bit}$ at around 4.6748 μm. Meanwhile, for S_2_ at l = 1.725*a*, the highest bandwidth with zero-dispersion flat band was obtained at about ∆λ = 2.9488 nm, however with a high average group index of ${\bar{n}}_{g}$ = 144 that leads to longer delaying time of 480 ps. By increasing l, the ultra-low group velocity can be acquired (high group index) with accomplishment delaying time of ns range (1.603 ns). Nevertheless, to store 1 bit, a long bit length of 11.3158 μm is obtained. Such an increase of bit length is due to the decrease of cavity density along the horizontal axis of waveguide, because each cavity serves a center of the stored data. Consequently, the proposed S_2_ SP-CS-SPCW is suitable for optical switching and high-speed optical modulator at this value of l. 

**Figure 4:**
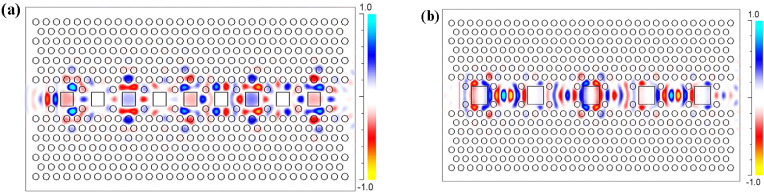
The magnetic field profile of pulse transmission inside the optimum structures for (**a**) S_1_ at l=1.25a and (**b**) S_2_ at l=1.725a.

**Figure 5:**
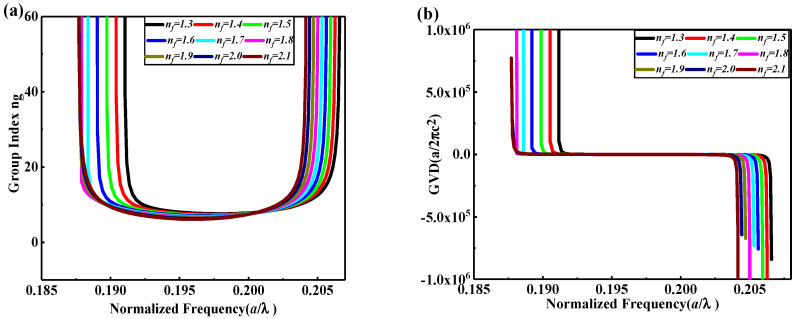
Slow-light properties of the optimum S1 SP-CS-SPCW for refractive indices of RTILs (n) varying from 1.3 to 2.1 at l=1.25a versus normalized frequency a/λ for (**a**) group index ($n_{g})$ and (**b**) group-velocity dispersion (GVD).

Therefore, we can consider S_2_ SP-CS-SPCW for the longest storage time and S_1_ SP-CS-SPCW for wider bandwidth and high transmission bit rate. Figure 4 shows the magnetic field (M-field) profile of pulse transmission inside the optimum structures for S_1_ at l=1.25a and S_2_ at l=1.725a. In Figure 4a, the field distribution spreads out the waveguide with strong coupling between successive slots. Additionally, periodic oscillations for the field contour are detected along the propagation path. In contrast, in Figure 4b, the localization is increased inside the slab with a longer time for making a hop from one cavity to another, which reflects the lower transmission bandwidth with the ultra-low group velocity. The packet compression and decompression may prove useful in high-speed communication and signal processing, including multiplexers. For further optimization and showing the effect of RTIL infiltrated, the square slots are assumed to be filled by ionic liquid whose reflective index is from 1.6 to 1.7 for optical communication wavelength [11]. The main factor for the theoretical calculations is the refractive index. So, we have done our calculations for low and high indices of RTILs’ ranges to meet wide applications. However, there are limited choices for chemically and physically stable, nontoxic liquids as optical materials, especially when the required refractive index is higher than 1.60. Commercially available high-index liquids are often based on harmful or toxic materials, such as liquids based on diiodomethane [27]. High-index RTILs were verified to be appropriate candidates, in this case, to be useful as index-matching liquids [27,28]. Some ILs, especially the early ones, are not stable and these ILs are to be avoided for applications. Many of the new-generation ILs are stable and can be used in the proposed structures. Figure 5 and Table 2 show the slow-light properties, $n_{g}$ and *GVD,* of the optimum S_1_ SP-CS-SPCW at l=1.25a with the refractive index of RTIL ($n_{f}$) varying from 1.3 to 2.1. It can be seen that the performance of the guided slow-light modes is significantly affected also by the refractive index of RTIL infiltrated. 

The guided modes of the slow light shift to the lower frequency area and the bandwidth increases when the refractive index of RTIL decreases slightly for $n_{f}<1.8$. The shift of mode frequency is physically understandable as the effective refractive index of the structure increases, so that the optical size of the structure increases and, thus, the frequency response scales to the low-frequency area. Table 2 shows the improvement of slow-light property and buffering parameters for the slot holes infiltrated with RTILs with varying $n_{f}$ from 1.3 to 2.1. Polymerized RTILs enhance the transmission rate through optical materials. From the optical properties of polymerized RTILs, it shows that the optimum FoM at high index polymerized RTILs of $n_{f}=1.8$ is 0.72402. For 6G networks, the transmission data rate can be 1–10 Tb/s [29,30]. As shown in Table 2, ultra-wide frequency bandwidth is obtained with a high bit rate of about 3.93629 Tb/s with a high $R_{b}$ of 3.93629 Tb/s, which is so high that they are never seen in the literature, and $L_{bit}$ is enhanced to be 4.2817 μm. PhCs are made of lossless materials [31,32]. However, in reality, all materials have some loss. So, the loss of PhCs could be a factor to be considered in their applications. Fortunately, due to the small size of optical integrated chips, the material loss can be ignored for available materials and, further, the structure losses can be reduced or eliminated through carefully designing the dispersion of the waveguides, explained in detail at [17,33,34,35]. Therefore, PhCs have great potential for information and communication technologies, including 6G wireless communication [31]. Their potential applications for micro-optical components include waveguides, micro-resonators, and photonic crystals, which are essential components in photonic integrated circuits (PICs). 

## 4. Implementation Considerations

Some restrictions can appear for practical realization of the buffer devices, such as dispersion and losses.

### 4.1. Dispersion 

One of the main factors restricting short-pulse light transmission is material dispersion that induces *GVD,* which causes pulse width expansion and induces phase modulation. Accordingly, the transmitted light pulse that carries the data bits turns out to be vastly distorted during the propagation path. Thus, the distortion compensation by the *GVD* is needed in the transmission [36]. For the compensation resolves, the designs with positive and negative GVDs are shown in Figure 5b. Additionally, the slow light transmitted in the waveguide, in general, is intrinsic dispersion. Henceforth, designing a wide zero-dispersion flat band is required. From Figure 5b, it can be observed that *GVD* is less than $10^{6\;}a/2\pi c^{2}$ in the near-zero-dispersion flat band, which is an adequate *GVD* because the value of *GVD* below $10^{6\;}a/2\pi c^{2}$ can be considered as low *GVD* [37]. Moreover, it is interesting to note that the *GVD* changes with negative and positive values in the operating frequency range, which is suitable for dispersion compensation applications.

### 4.2. Losses 

Overall, there are two types of losses: Material and structure losses. By choosing the appropriate materials, the material loss can be ignored or avoided [38,39]. The structure loss for the buffer devices or any kind of waveguide comes from two aspects. The first one is the coupling loss at the input and output of structures or devices. The second one is the propagation losses over the buffer device region. To keep the bandwidth and the advantages of buffer devices, it is suitable to employ traditional Si waveguide as a transmission intermediate devoid of any loss and use buffer devices as active elements [35]. Now, the coupling loss can be considered as a frequently resolved issue [35]. Propagation losses in the buffer device region can be divided into intrinsic and extrinsic losses. For the practical realization of the buffer devices, the dispersion curves of waveguide modes would be below the light line, because the modes positioned above the light line are highly loss, and the modes under the light line are intrinsically lossless [25]. All the calculations have been done under light lines in the proposed structures. Professionally, both dispersion and loss issues are controlled by specific designs of the proposed structures, since the advantageous characteristic of slow light is a collaborative result through the broadening of pulse and loss [40].

The proposed SP-CS-SPCW provides an abundantly advanced value of the slow-light property (*NDBP*) and all optical buffering parameters compared with all other structures, reported previously in the literature, as shown in Table 3. In comparison with other works, we found that our work has high *NDBP* and the largest transmission bit rate. High-speed communication and signal processing are interoperating fields. To transmit any signal over a wide range (from baseband to bandpass), the modulation technique is used. The analog data in the form of voice, image, or video captured is converted into a digital format, which is further processed for long-range communication. So, processing of digital data requires signal processing, and transmitting this data over long-range requires wireless or fiber communication. Digital signal processing is used because it is more immune to noise and is secured, cheap, and easy to process. Generally, the processed digital data is encoded for sources and channels to give protection and compression simultaneously. The proposed SP-CS-SPCW can find the application in this area, as high-speed optical communication systems require signal processing, e.g., signal modulation and encoding/decoding, while signal processing generally requires data buffering. So, the proposed structure is useful. 

## 5. Conclusions

Slow-light low dispersion and buffering properties in the coupled-slot slot and coupled-slot slab cavities’ waveguide were studied in detail by appropriately modifying the square air slot length. In the proposed SP-CS-SPCW, we obtained a flat band of zero dispersion with the combined constant group index ranging from 8.26 to 481. Moreover, this variation is simultaneous with the changing of delaying time from 30.5 ps to a long delay time of 1603.3 ps. Light propagating by long delaying time and high group index affords the opportunities for advancing photonic integration with permitting compact switches of ultra-small electro-optical devices. Furthermore, the smallest physical length of each storage bit was obtained as $L_{bit}$ = 4.2817 μm, which is ultra-small. Additionally, in the further optimized case, we infiltrated the slots with RTILs, which boosted the bandwidth to 126.09 nm. From the optical properties of polymerized RTILs, they seem to be promising materials for the fabrication of infrared photonic elements. Their potential applications for micro-optical components include waveguides, micro-resonators, and photonic crystals, which are essential components in photonic integrated circuits (PICs). Finally, the proposed SP-CS-SPCW can find application in this area, as high-speed optical communication systems require signal processing, e.g., signal modulation and encoding/decoding, while signal processing generally requires data buffering. So, the proposed structure is useful. Moreover, it is interesting to note that the *GVD* changes with negative and positive values in the operating frequency range, which is suitable for dispersion compensation applications. 

## Figures and Tables

**Table 1:** Slow-light parameters and buffering capability under different values of l for S_1_ and S_2_.

Structure	l/a	${\bar{n}}_{g}$	∆U	∆λ (nm)	*NDBP*	$T_{s}\;(\mathrm{ps})$	C(bit)	$L_{bit}\left(\mu m\right)$	$R_{b}(Tb$ /s)
S_1_	1.225	9.5	0.01343	104.81	0.64237	31.667	207.215	4.8259	3.2718
	1.25	10	0.01327	102.78	0.66313	33.333	213.912	4.6748	3.2087
	1.275	10.4	0.01239	95.689	0.64205	34.667	207.112	4.8283	2.9872
	1.3	10.72	0.01167	89.499	0.61899	35.733	199.673	5.0082	2.7939
	1.325	11	0.01139	86.761	0.61573	36.667	198.621	5.0347	2.7085
	1.35	11.68	0.0108	81.927	0.61736	38.933	199.149	5.0214	2.5576
S_2_	1.7	137	0.00037	2.8196	0.24922	456.67	80.393	12.4389	8.8 × 10^−5^
	1.725	144	0.00039	2.9488	0.27395	480	88.372	11.3158	9.2 × 10^−5^
	1.75	173	0.00024	1.7842	0.19914	576.67	64.238	15.5671	5.6 × 10^−5^
	1.775	198	0.00019	1.4281	0.18243	660	58.847	16.9931	4.5 × 10^−5^
	1.8	204	0.00018	1.3955	0.18367	680	59.247	16.8785	4.4 × 10^−5^
	1.825	242	0.00013	1.0153	0.15852	806.67	51.136	19.5559	3.2 × 10^−5^
	1.85	313	8.4 × 10^−5^	0.6354	0.12831	1043.3	41.391	24.16	2 × 10^−5^
	1.875	348	6.4 × 10^−5^	0.4836	0.10858	1160	35.026	28.55	1.5 × 10^−5^
	1.9	481	3.5 × 10^−5^	0.267	0.08286	1603.3	26.73	37.4107	8.3 × 10^−6^

**Table 2:** Slow-light parameters and buffering capability at different refractive indices of RTILs ($n_{f}$ from 1.3 to 2.1).

Structure	$n_{f}$	${\bar{n}}_{g}$	∆U	∆λ (nm)	*NDBP*	$T_{s}\;(\mathrm{ps})$	C(bit)	$L_{bit}\left(\mu m\right)$	$R_{b}(T$ b/s)
S_1_	1.3	9.33	0.01387	108.13	0.65086	30.5	209.956	4.7629	3.37549
	1.4	9.15	0.01417	110.74	0.65375	29.9	210.888	4.7419	3.45718
	1.5	8.97	0.01447	113.36	0.65602	29.067	211.619	4.7255	3.53878
	1.6	8.72	0.01481	116.47	0.65523	29.433	211.366	4.7311	3.63588
	1.7	8.83	0.01556	122.66	0.69878	29.667	225.414	4.4363	3.82923
	1.8	8.9	0.01595	126.09	0.72402	28.8	233.553	4.2817	3.93629
	1.9	8.64	0.01521	120.15	0.66974	28.233	216.046	4.6287	3.75079
	2.0	8.47	0.01471	116.27	0.63537	27.533	204.959	4.879	3.62974
	2.1	8.26	0.01425	112.64	0.60028	30.5	193.637	5.1643	3.51642

**Table 3:** Comparison between current work and previous literature.

Refs.	*NDBP*	$R_{b}(T$ b/s)	C(bit)	$L_{bit}\left(\mu m\right)$
Current work	0.72402	3.93629	233.553	4.2817
[22]	0.14	Non	Non	Non
[41]	0.221	Non	Non	Non
[42]	0.6858	2.3125	221.2	4.5202
[43]	0.31	Non	Non	Non
[20]	0.4661	Non	143.3	6.95
[37]	0.6456	Non	Non	Non
